# Support for Mandatory COVID-19 Vaccines for 5- to 11-Year-Old Children: Cross-sectional study of Omani mothers

**DOI:** 10.18295/squmj.3.2024.018

**Published:** 2024-05-27

**Authors:** Salah Al Awaidy, Faryal Khamis, Thamra Al Ghafri, Abdallah Badahdah

**Affiliations:** 1 Health Affairs, The Royal Hospital, Muscat, Oman; 2 Department of Medicine, The Royal Hospital, Muscat, Oman; 3 Directorate General of Health Services, Ministry of Health, Muscat, Oman; 4 School of Psychology, Sociology and Rural Studies, South Dakota State University, Brookings, USA

**Keywords:** COVID-19, Mandatory Vaccine, Vaccine Hesitancy, Children, Oman

## Abstract

**Objectives:**

This study aimed to investigate the variables that influenced a sample of Omani mothers’ support for mandatory COVID-19 vaccines for children. The vaccination against COVID-19 averted millions of fatalities during the COVID-19 pandemic. Nevertheless, a considerable number of parents and caregivers opposed mandating COVID-19 vaccines for children.

**Methods:**

This cross-sectional study was conducted at several healthcare facilities in Oman using a structured questionnaire between February and March 2022. Univariable and multivariable logistic regression models were used to analyse the data.

**Results:**

A total of 700 Omani mothers (response rate = 73.4%) who had children aged 5–11 years old were included. The median age of the mothers was 38 ± 5.19 years. The results of multivariable logistic regression were generally consistent with those of the univariable analysis except for age (odds ratio [OR] = 1.06, 95% confidence interval [CI]: 0.58–1.93; *P* = 0.86) and income (OR = 1.09, 95% CI: 0.58–2.03; *P* = 0.79). Mothers who were vaccine hesitant (OR = 9.82, 95% CI: 5.27–18.28; *P* <0.001), tested positive for COVID-19 (OR = 3.25, 95% CI: 1.80–5.86; *P* <0.001) and had one or two doses of COVID-19 vaccines (OR = 5.41, 95% CI: 2.92–10.03; *P* <0.001) were more likely to refuse mandating COVID-19 vaccines for children 5–11 years old.

**Conclusion:**

Mothers who were vaccine hesitant, tested positive for COVID-19 and had one or two doses of COVID-19 vaccines were more likely to oppose mandatory COVID-19 vaccines for young children. The findings should aid public health authorities in designing future childhood vaccine literacy programmes with specific attention to some subgroups in Oman to help reduce opposition to vaccines in future pandemics among mothers.

**Advances in Knowledge**

- *This study found out that vaccine hesitancy, testing positive for COVID-19 and 1*–*2 doses of vaccines against COVID-19 significantly influenced support for mandatory COVID-19 vaccines among 5- to 11-year-old children in Oman.*

**Application to Patient Care**

- *This study’s results may aid public health authorities in designing future childhood vaccine literacy programmes with specific attention to some subgroups in Oman to help reduce opposition to vaccines in future pandemic among mothers.*
- *This study can help to effectively implement evidence-based mandatory COVID-19 vaccination initiatives among children.*
- *These findings may enable comprehensive health communication strategies in future pandemics that promote the uptake of childhood vaccinations.*

The COVID-19 vaccines reduce the risk of both infection and transmission of the virus among children.^1^ A small proportion of children experienced severe illnesses due to COIVD-19 that required treatment, and a few have died as a result of the disease.^2^ Specifically, children with underlying comorbidities such as obesity, heart, kidney or liver disease and cancer are at a greater risk of developing severe COVID-19 disease. Children with COVID-19 are susceptible to developing long-COVID, which is characterised by symptoms such as fatigue, brain fog and shortness of breath.^3^ Hence, vaccinating children against COVID-19 should reduce the possibility of developing COVID-19-related health issues and interfering with their education and social activities.^4^

A large body of research has explored caregivers’ intention to vaccinate their children against COVID-19.^5^–^7^ It shows that support for vaccination of children against COVID-19 is complex and influenced by several factors such as concerns about vaccine safety, belief in conspiracy theories, effectiveness of the vaccines and caregivers’ sociodemographic variables, including age, income and education. Support for COVID-19 vaccines for children, however, waxed and waned throughout the pandemic.^8^–^11^ A study conducted from 2021–2022 found that parental intent to vaccinate children against COVID-19 declined over 3 months, but reverted to baseline after 6 months.^11^

In the fight to stop or slow the spread of COVID-19, several governments, schools, healthcare entities and private businesses around the world mandated COVID-19 vaccination.^12^^,^^13^ Nevertheless, debates erupted during the pandemic regarding the legality, ethics and effectiveness of mandated COVID-19 vaccines for some populations, including children.^14^–^17^

Although studies in this regard indicate that vaccination rates were low among young children, little research has been done to explore caregivers’ support for mandatory COVID-19 vaccines, especially in the Arab world.^18^–^23^ The current study presents survey data gathered in 2022 and aimed to investigate the attitudes of Omani mothers towards mandating COVID-19 vaccines for children 5–11 years old. To better understand the factors that correlate with attitudes, several variables including age, income, employment status, educational attainment and vaccine hesitancy were examined. The findings of the present study will provide public health in Oman with data to better prepare for future pandemic-related vaccination campaigns.

## Methods

This cross-sectional study was conducted in Muscat governorate, including Muscat (the capital of Oman) which is the largest of the 11 governorates with an estimated 1,302,440 inhabitants. The Directorate General of Health Services in Muscat governorate houses 30 primary healthcare centres, 2 polyclinics and 3 hospitals. Mothers of children between the ages of 5 and 11 years were recruited using a convenience sampling method. Only Omani mothers aged 18 years and older with children between the ages of 5 and 11 years were enrolled in the study. The participants were approached during their visits to 7 primary care centres in Muscat governorate by 14 family physicians between February and March 2022. The 14 research assistants, all family physicians, were recruited and trained to administer a face-to-face Arabic questionnaire.

To determine the sample size, it was assumed that 50% of the mothers were hesitant about their views. Hence, a minimum of 550 participants were needed with an interval of confidence of 99% (z value = 2.58), a margin of error of 5% (delta value = 0.05) and a participation refusal rate of 20%.

Sociodemographic data were collected from the participants, including age, household income, educational attainment, employment status and the number of children aged 5–11 years. They were also asked about COVID-19 infections, COVID-19 vaccination status and who the decision-maker in the family is concerning vaccinating children against COVID-19. The age of the participants was dichotomised based on the median value (median = 38 years) into ‘38 and younger’ and ‘39 and older’. Household income was measured by an open-ended one item and then divided into ‘≤4,679 USD (1,802 Omani Rials)’ and ‘≥4,680 USD (1,724 Omani Rials)’ groups. A total of 6 response categories that ranged from 1 (did not attend school) to 6 (postgraduate degree) were used to assess educational level. The educational level was then divided into ‘high school or less’ and ‘college degree or higher’ categories. A binary item (employed/not employed) was used to report employment status. The participants were also asked regarding the history of their COVID-19 test results, with the response categories being ‘positive’, ‘negative’, ‘not sure/do not know’. The COVID-19 vaccination status was assessed by 1 question that asked if they had received a vaccination against COVID-19 or not. If they had, the participant was asked to choose from two subcategories: ‘one dose/two doses’ and ‘two doses and a booster’. Finally, the participants reported on who would make the final decision about whether their children should be vaccinated against COVID-19; 4 response options were given, including ‘me only’, ‘my husband only’, ‘both my husband and I’ or ‘do not know’. Before the actual study was conducted, the study questionnaire was pilot-tested using a sample of 25 mothers with children 5–11 years old. Appropriate changes were made including adding missing words and the removal of 2 items.

Support for mandatory COVID-19 vaccines for children 5–11 years old was measured by a single item: ‘COVID-19 vaccines should be compulsory in Oman for children of ages 5–11.’ The item was followed by a 5-point Likert scale, with 1 representing strongly disagree and 5 indicating strongly agree. To establish a clear picture of the participants’ support for mandating COVID-19 vaccines, the ‘not sure’ responses were removed from the analysis. Then, the item was averaged and dichotomised using the median as a cut-off point into supportive (strongly agree and agree) and not supportive (strongly disagree and disagree) of mandatory COVID-19 vaccines.

For the assessment of vaccine hesitancy, the participants were asked to respond to a 4-item scale, using a 5-point Likert-type format that ranged from 1 (strongly disagree) to 5 (strongly agree). Items were adopted from previous studies with some modifications.^24^^,^^25^ Examples of these items were ‘I do not trust that the COVID-19 vaccines can protect children from COVID-19 disease’ and ‘children COVID-19 vaccines are effective’. An exploratory factor analysis using principal axis factoring with the Promax rotation (k = 4) showed a one-factor solution that explained 58% of the variance in the data (α = 0.85). After 1 item was reverse coded, all items were averaged, as higher scores represented lower levels of hesitancy and then dichotomised using the median score (median = 3.0) as a cut-off point. Hence, the participants who scored equal to or above the median were classified as ‘less hesitant’.

Descriptive statistics, including mean, standard deviations, frequency and percentages, were used to describe the study variables. To explore the factors associated with support for mandating COVID-19 vaccines for children 5–11 years old, univariable and multivariable logistic regression models were used. All statistical analyses were performed using Statistical Package for Social Sciences (SPSS), Version 29 (IBM Corp., Armonk, New York, USA).

Univariable binary logistic regression analyses were performed to assess the association between sociodemographic variables (age, income, educational level and employment status) and COVID-19-related factors (vaccine hesitancy, COVID-19 vaccination status and COVID-19 test results) and the dependent variable of opposing mandatory COVID-19 vaccines for children 5–11 years old. Variables with *P* <0.10 in the univariate analysis were included in the multivariate logistic regression analysis to identify variables associated with the dependent variable. The odds ratio (OR) values and their 95% confidence intervals (CI) were calculated. The Hosmer and Lemeshow Chi-squared test was conducted to evaluate the model fit of the multivariable logistic regression analysis. A *P* value of <0.05 was deemed statistically significant.

Informed consent was obtained from all the participants. Participation was voluntary and anonymous; the participants received no compensation. The study was approved by the Regional Study Approval and Ethical Review Committee (MoH/CSR/22/25452) at the Directorate General of Health Services in Muscat governorate, Ministry of Health, and conducted per the Declaration of Helsinki.

## Results

A total of 954 eligible mothers were approached of which 700 completed the surveys (response rate = 73.4%). The participants had a median age of 38 ± 5.19 years (range: 25–53 years) with a most (75%) having 1–2 children. Almost half of these children (43.9%) were girls. Most of the mothers (73%) had a college degree or higher, and 70.8% were working full-time, mostly in the public sector (84.5%). Slightly less than half (46.9%) made ≤4,679 USD. A small number of the participants were not employed (29.2%). Half of the sample tested positive for COVID-19, 42.7% tested negative and the rest (7.4%) were not sure or did not know. Almost all (92.9%) mothers reported that they and their husbands would make a joint decision regarding vaccinating their children against COVID-19. As for the outcome variable, after the participants who were not sure (n = 92) were removed from the logistic regression analyses, only a small percentage (25.3%) were supportive of mandatory vaccination, while the majority (74.4%) were against it [Table 1].

The results of the univariable binary logistic regression analysis showed that mothers ≤38 years (OR = 2.35, 95% CI: 1.62–3.41; *P* <0.001), low-income mothers (OR = 2.98, 95% CI: 1.90–4.67; *P* <0.001), vaccine-hesitant mothers (OR = 11.73, 95% CI: 7.56–18.19; *P* <0.001), those who tested positive for COVID-19 (OR = 2.75, 95% CI: 1.87–4.06; *P* <0.001) and those who got one or two doses of the COVID-19 vaccines (OR = 2.75, 95% CI: 1.87–4.06; *P* <0.001) were significantly associated with higher odds of rejecting mandatory COVID-19 vaccines for 5–11-year-old children. Finally, support for mandatory vaccination was not related to educational status (*P* = 0.11) or employment status (*P* = 0.25) [Table 2].

As for the multivariable logistic regression, the Hosmer and Lemeshow test indicated a good fit of the data (χ^2^ = 9.82 [8]; *P* = 0.28). The logistic regression model was statistically significant (χ^2^ [5] = 189.52; *P* <0.001) and explained 54.0% (Nagelkerke R^2^) of the variance in opposition to mandatory vaccines and correctly classified 85.8% of cases. The results were generally consistent with that of the univariable analysis except for age (OR = 1.06, 95% CI: 0.58–1.93; *P* = 0.86) and income (OR = 1.09, 95% CI: 0.58–2.03; *P* = 0.79). That is, vaccine-hesitant mothers (OR = 9.82, 95% CI: 5.27–18.28; *P* <0.001), those who tested positive for COVID-19 (OR = 3.25, 95% CI: 1.80–5.86; *P* <0.001) and those who had one or two doses of the COVID-19 vaccines (OR = 5.41, 95% CI: 2.92–10.03; *P* <0.001) were associated with refusal to mandate COVID-19 vaccines for children 5–11 years old.

## Discussion

In this cross-sectional study, data from Omani mothers were collected to explore the effect of their age, income, education, employment status, vaccine hesitancy, infection with COVID-19 and COVID-19 vaccination on their support for mandatory COVID-19 vaccines for children 5–11 years old. The results of the multivariable logistic regression analysis suggested that vaccine-hesitant mothers, those who tested positive for COVID-19 and those who had one or two doses of the COVID-19 vaccines were associated with a refusal to support mandatory COVID-19 vaccines.

The current study showed that 74.4% of the mothers rejected the idea of mandating COVID-19 vaccines for children, while the minority (25.3%) were in favour of it. Although most studies exploring COVID-19 vaccination focused on parents rather than mothers, the current study’s finding is similar to a study in Jordan where 77.6% of the parents opposed mandating COVID-19 vaccines for children.^26^ Other studies, however, showed higher rates of support for mandatory vaccines, including a study of parents of children aged 2–15 years in India that found 81% of the parents endorsed COVID-19 mandatory vaccines for children.^27^ In Poland, 44.4% of the parents believed that vaccinations should be mandatory, while in Germany, this was 31%.^23^^,^^28^ A similar result was obtained from parents in New York City, where a study found that 44.3% of the parents supported school-based vaccine mandates for students.^28^ When mandatory COVID-19 vaccination was linked to school attendance, 44% of the caregivers stated that vaccines should never be mandated.^13^

One potential explanation for the strong opposition to mandatory vaccination in this study is that the participants may have had low levels of COVID-19 vaccine literacy. Work on the vaccination of children during and before COVID-19 showed a link between health literacy and the likelihood of childhood vaccination.^29^–^31^ Hence, it is important to provide caregivers with accurate information for future vaccines and to increase levels of trust in the health establishment.

The study found that mothers who tested positive for COVID-19 and those who received COVID-19 vaccines were associated with higher odds of rejecting mandatory COVID-19 vaccines for their children. One plausible explanation is that these mothers had some concerns regarding the efficacy and safety of the vaccines, despite almost all of them having received COVID-19 vaccines, and the belief that children may obtain immunity through infection. Another interpretation is that the mothers were hesitant adopters. In other words, despite the majority of them having been vaccinated, they exhibited vaccine-hesitancy. ^32^

This study is subject to some limitations. First, as with all self-reported surveys, there is a risk of potential bias; the participants’ responses may have been affected due to the presence of the interviewers. They might have exaggerated some of the information provided, such as their vaccination status, due to social desirability. Additionally, the study utilised a convenience sample of mothers attending health centres in one governorate, which limits the generalisability of the findings. Third, the survey was conducted between February and March 2022, and the perceived vaccine benefits and risks could have changed over time, especially with the emergence of variants causing less severe disease. Fourth, the study results cannot be applied to the Omani community at large as most of the women who took part in the study have college degrees. Finally, some other variables might have influenced the mothers’ support for COVID-19 mandatory vaccines that were not included in this study, such as belief in conspiracy theories and perceived severity of the disease.

## Conclusion

This study provides an insight into attitudes towards mandatory COVID-19 vaccination for young children among mothers before the start of a vaccination campaign targeting children aged 5–11. Mothers who exhibited vaccine hesitancy, tested positive for COVID-19 and had received one or two doses of the COVID-19 vaccine were more inclined to oppose compulsory vaccination of small children with COVID-19. While new variants of COVID-19 continue to emerge amid the waning of COVID-19 vaccine-induced protection, evidence-based mandatory childhood vaccination policies against COVID-19 and future pandemics, that do not impose unnecessary burdens on parents, are desirable.

## Figures and Tables

**Table 1:** Characteristics of Omani mothers who had children aged 5–11 years old (N = 700)

Characteristic*	n (%)
**Median age in years ± SD (range)**	38 ± 5.19 (25–53)
**Monthly income in USD (n = 497)**	
≤4,679	233 (46.9)
≥4,680	264 (53.1)
**Education (n = 699)**	
College degree or higher	510 (73.0)
High school or less	189 (27.0)
**Employment status (n = 695)**	
Working	492 (70.8)
Not working	203 (29.2)
**COVID-19 experience**	
Tested positive	349 (49.9)
Tested negative	299 (42.7)
Not sure/do not know	52 (7.4)
**Vaccination status (n = 694)**	
One or two doses	443 (63.8)
Two doses and a booster	251 (36.2)
**COVID-19 vaccine decision-maker (n =688)**	
Mother or father only	49 (7.1)
Both mother and father	639 (92.9)

SD = standard deviation; USD = United States Dollar.

* Frequencies may not add up to the total sample size due to missing responses.

**Table 2:** Opposition to mandating COVID-19 vaccination of children 5–11 years old by vaccine hesitancy, vaccination status, COVID-19 test result and demographic variables

Variable	Univariable model, OR (95% CI)	*P* value	Multivariable model, OR (95% CI)	*P* value
**Age in years (ref: ≥39)**				
≤38	2.35 (1.62–3.41)	≤0.01	1.06 (0.58–1.93)	0.86
**Income (ref: ≥4,680 USD)**				
≤4,679	2.98 (1.90–4.67)	≤0.01	1.09 (0.58–2.03)	0.79
**Education (ref: college degree or higher)**				
High school or less	1.42 (0.93–2.18)	0.11		
**Employment (ref: working)**				
Not working	1.27 (0.84–1.92)	0.25		
**Vaccine hesitancy (ref: low hesitancy)**				
High hesitancy	11.73 (7.56–18.19)	≤0.01	9.82 (5.27–18.28)	<0.001
**COVID test (ref: tested negative)**				
Tested positive	2.75 (1.87–4.06)	≤0.01	3.25 (1.80–5.86)	<0.001
**Vaccination status (ref: two doses and boosted)**				
One or two doses	2.75 (1.87–4.0)	≤0.01	5.41 (2.92–10.03)	<0.001

USD = United States Dollar.
